# Treatment of stasis dermatitis using aminaphtone: a case series

**DOI:** 10.1186/1752-1947-4-295

**Published:** 2010-08-31

**Authors:** José Maria Pereira de Godoy

**Affiliations:** 1 The Angiology and Vascular Surgery Service of the Medicine School in São José do Rio Preto, FAMERP, Rua Floriano Peixoto 2950, São Paulo, Zip code 15010-020, Brazil

## Abstract

**Introduction:**

Stasis purpura is a common finding in clinical practice and is related to vascular alterations.

**Case presentation:**

Four randomly-selected, Caucasian patients (a 45-year-old woman, a 26-year-old man, a 51-year-old man and a 56-year-old woman) were treated with aminaphtone for approximately one year. For all patients, the brown patches - a sign of stasis purpura - disappeared without the appearance of new lesions within this period.

**Conclusion:**

Aminaphtone is a novel proposal in the treatment of stasis purpura when capillary fragility is identified.

## Introduction

Chronic venous disease (CVD) is common with manifestations that include varicose veins, skin changes such as dermatitis, hyperpigmentation, lipodermatosclerosis, and chronic leg ulcers [1]. Venous stasis disease is involved in 70 to 90 percent of all lower-extremity ulcers treated. Venous hypertension, due to inadequate venous return associated with a defective valvular system, is the main culprit [2]. Lipodermatosclerosis is an indurated plaque in the medial malleolus that can, at times, be quite tender and painful [1,3].

Venous dermatitis is often the first manifestation of venous insufficiency and needs to be addressed promptly [3]. Hemosiderin and/or melanin have been considered responsible for the brown pigmentation [4]. On histopathologic evaluation, dermal melanocytes containing melanin and incontinence of melanin pigment were observed, which suggests that melanin pigment from the epidermis may contribute to cutaneous pigmentation in stasis dermatitis [4]. There is a scarcity of therapies to treat stasis dermatitis recorded in the literature; however, the use of substances that control capillary fragility may be useful to control bleeding [5]. Capillary fragility is a condition where the smallest blood vessels, the capillaries, become weak and may rupture, leaking blood into the surrounding tissues. This bleeding is identified initially by small reddish punctiform spots or petechiae which become brown with time.

My aim in this case report is to describe the long-term use of aminaphtone in the treatment of stasis purpura when capillary fragility is identified.

## Case presentation

### Case 1

A 45-year-old Caucasian woman presented with brown patches on the lower third of her leg, which had been present for three years prior to our interview. She reported no other clinical complaints. She described that the lesions started as small reddish spots which became brown over time. A physical examination confirmed that the brown patches were associated with reddish punctiform petechiae. Additionally, clinical, etiology, anatomic and pathophysiology (CEAP) 1 telangiectasia was observed. A clinical diagnosis of stasis purpura was reached and she was medicated with 75 mg aminaphtone, twice daily for one year, resulting in a complete remission of the lesions. No new punctiform lesions appeared during a three-year follow-up period.

### Case 2

A 26-year-old Caucasian man presented with a history of brown patches on both legs that had started two years previously with the appearance of reddish spots. On physical examination, the presence of brown patches together with reddish spots was confirmed, predominantly on the distal third of his legs including his ankle region. He did not suffer from varicose veins or telangiectasias. The clinical diagnosis was stasis purpura and he was medicated with 75 mg aminaphtone, twice daily until the lesions disappeared completely, which took about 11 months. No new lesions appeared during a three-year follow-up period.

### Case 3

A 51-year-old Caucasian man presented with a history of brown spots on his lower leg. These had appeared as reddish spots approximately two years previously and became brown over time. On physical examination, brown patches together with reddish punctiform petechiae were found on the lower third of his leg up to the ankle region. CEAP C1 telangiectasia was observed in both of his legs. A clinical diagnosis of stasis purpura was made and he was medicated with 75 mg aminaphtone, twice daily until the lesions disappeared, which took about 13 months. No new lesions appeared during a one-year follow-up period.

### Case 4

A 56-year-old Caucasian woman presented with pain in her legs, progressive brown patches and varicose veins over a period of four years. On physical examination, dilated veins, edema, brownish patches and reddish punctiform spots were observed, involving the lower third of her limbs and ankle regions. A Doppler echogram was performed that demonstrated saphenous vein insufficiency and collateral varicose veins. She was submitted to varicose vein surgery for resection of her saphenous vein and collateral varicose veins. A clinical diagnosis of stasis purpura was made and she was prescribed 75 mg aminaphtone until the brown patches disappeared, which took about 14 months. No new lesions have appeared over the last two years.

## Discussion

I describe the clinical improvement of stasis purpura with aminaphtone. Aminaphtone is a common name for compound 2-hydroxy-3-methyl-1,4-napthohydroquinone-2-p-aminobenzoate. To the best of my knowledge, this approach has not been described in the literature and thus this is a novel therapeutic option.

One hypothesis is that a possible cause of stasis purpura is capillary fragility and by improving this fragility it is possible to treat and even eliminate this form of purpura. Initially, the appearance of new punctiform spots is controlled and then there is a gradual reduction in the size of the brown patches.

An important aspect of therapy is the duration of treatment. This has proven to be about one year, after which time a total elimination of the brown patches is evidenced. All patients were followed up every two months, when control of the appearance of new lesions and a reduction of existing lesions was observed. It is important that patients are made aware of the expected duration of the treatment as they will want to see immediate results. Photographic documentation is recommended for clinical evolution control purposes. The use of aminaphtone for short periods, such as one month, does not seem to be efficacious, and so the duration of treatment is the determining factor. The only side effect reported by a minority of the patients was gastric irritation.

The lack of efficacious therapeutic alternatives with respect to this disease drew my attention. Additionally, younger patients (20 to 30 years old), without evidence of clinical varicose veins (CEAP 0 and 1) are affected. This suggests that capillary fragility plays an important role in the development of this type of lesion. Even so, patients with more advanced chronic venous insufficiency (CEAP 5 and 6) can present with lesions and might benefit from this treatment. This treatment interferes in the physiopathology of the disease. Stasis dermatitis has been observed in some CEAP 4, 5 and 6 patients, frequently involving the entire circumference of the lower third of the leg, including regions unaffected by venous backflow. This observation suggests changes in capillary permeability.

After a few days of treatment, new reddish petechiae did not appear suggesting that the capillary fragility was controlled. The decision to maintain treatment over a prolonged period was reached due to the lack of information about how long this drug maintains control of capillary fragility and to evaluate the final result. This case report presents the results of four randomly-chosen patients in order to illustrate the approach; however, more than 40 patients have been treated and followed up in this period and all had similar results.

Currently, I prescribe aminaphtone daily for a period of two months and then in alternate weeks. There is a necessity to establish the best treatment regimen to determine for how long aminaphtone is necessary. Some patients have attained control without the appearance of petechiae for more than three years after the cessation of the drug, suggesting long-term control. The use of aminaphtone to control nose bleeds has been observed for more than three years after the suspension of the drug in the cases of patients that had been restricted from participating in social activities due to the bleeding [5]. This suggests that after the initial control of capillary fragility, long-term or definitive maintenance is obtained. Aminaphtone has also been utilized in the control of idiopathic cyclic edema [6].

As what I have described is a series of case reports, further controlled studies, including randomized double-blind controlled trials, which aim to reproduce the results and define the optimal time and efficacy rate of treatment are necessary.

## Conclusions

Aminaphtone may be a novel option in the long-term treatment of stasis purpura when capillary fragility is identified.

## Competing interests

The author declares that they have no competing interests.

## Consent

This case series was approved by the local ethics committee (protocol n04248/2009). Written informed consent was obtained from the patients for publication of this case report and any accompanying images. A copy of the written consent is available for review by the Editor-in-Chief of this journal.
